# Spontaneous cervical artery dissection: is it really a connective tissue disease? A comprehensive review

**DOI:** 10.3389/fneur.2023.1241084

**Published:** 2023-10-11

**Authors:** Muhammed Enes Gunduz, Ramanathan Kadirvel, David F. Kallmes, Alessandro Pezzini, Zafer Keser

**Affiliations:** ^1^Department of Neurology, University of Massachusetts Chan Medical School, Worcester, MA, United States; ^2^Department of Neurosurgery, Mayo Clinic, Rochester, MN, United States; ^3^Department of Radiology, Mayo Clinic, Rochester, MN, United States; ^4^Department of Clinical and Experimental Sciences, Neurology Clinic, University of Brescia, Brescia, Italy; ^5^Department of Neurology, Mayo Clinic, Rochester, MN, United States

**Keywords:** cervical artery dissection, skin biopsy, connective tissue disorder, genetics, stroke

## Abstract

**Background:**

Spontaneous cervical artery dissection (sCeAD) is an important cause of stroke in young adults. The underlying pathophysiology remains unclear, without validated biomarkers to identify subjects at risk. Previous studies suggested the role of abnormalities in the connective component of the arterial wall.

**Purpose:**

To assess dermal ultrastructural aberrations of connective tissue by skin biopsy and genetic variations in sCeAD patients.

**Method:**

We searched the PubMed and Scopus databases until August 2023 with PRISMA guidelines. Original articles assessing skin biopsy in sCeAD patients were included. Two reviewers independently conducted the screening.

**Findings:**

We included 16 studies compromising 459 patients. Thirteen studies assessed ultrastructural changes and found aberrations of collagen and elastic fibers, described as irregular contours and calibers of collagen fibrils, composite flower-like fibrils, fragmented moth-eaten elastin, and microcalcifications, cumulatively in 50.5% of patients. Seven studies showed no causative mutations in collagen type I, III, V, or elastin genes. One study showed linkage between connective tissue alterations and mutation on chromosomes 15q2 and 10q26 using genome-wide linkage analysis, while another study found significant copy number variant enrichments in genes involved in extracellular matrix (COL5A2/COL3A1/SNTA1) and collagen fibril organizations (COL5A2/COL3A1). Finally, differential expression of extracellular proteins was linked to connective tissue disorder in patients with recurrent sCeAD using a quantitative proteomics approach.

**Conclusion:**

Current literature supports the hypothesis that an underlying, subclinical connective tissue disorder, likely genetically determined, may predispose to arterial wall weakness and sCeAD. Further studies with larger sample sizes and robust methodology are needed to better define the role of connective tissue in sCeAD pathogenesis.

## 1. Introduction

Being responsible for up to 25% of brain infarcts in young adults, cervical artery dissection (CeAD), a clinical condition biologically characterized by bleeding within the wall of the cervical arteries, is the most common cause of stroke at young age (1).

The craniocervical arterial wall consists of three layers: (i) the tunica intima, the innermost layer with endothelial cells; (ii) the tunica media, a thick muscular middle layer that is composed of vascular smooth muscle cells and extracellular matrix (ECM); and (iii) the tunica adventitia, the outermost layer that is an ECM coating (2). The two major components of the ECM are elastic fibers and collagen fibers. Elastic fibers comprise a diverse range of ECM species, among which elastin is the most represented. Elastin is attributed to providing distensibility in the vessels and distributing stress onto collagen. Collagen fibers are comprised of bundles of collagen fibrils, which are formed from collagen triple helix bundles (each collagen triple helix is made up of three collagen chains). In arteries, collagen is the greatest facilitator of the contractile changes that occur and is attributed to defining the stiffness of vessels. CeAD is a sudden tear most commonly within the intima with subsequent bleeding into subintimal space (3). This tearing results in the separation of the vessel wall and allows blood to flow into to intimal layer of the vessel, thus resulting in a false lumen and intramural hematoma, which may cause significant stenosis or occlusion of the artery and may lead to a transient ischemic attack or ischemic stroke (4–6). Most CeADs are either spontaneous (sCeAD) or occur in settings of mild non-penetrating trauma. Unfortunately, the underlying pathophysiology of sCeAD remains largely unknown, and it is unclear why some people develop the disease while most of the population does not.

Several risk factors, such as hypertension and migraine, have been associated with increased risk of CeAD (7). On the other hand, known nosographic entities, such as hereditary connective tissue disorders (HCTD, i.e., Marfan’s syndrome, Ehlers-Danlos syndrome type IV or Loeys-Dietz disease), have been documented to cause sCeAD in some affected individuals (7–10), while recent studies showed subtle or subclinical connective tissue aberrations in patients with sCeAD (11). Isolated mild, clinically detectable, connective tissue abnormalities in skeletal, ocular, and skin systems (i.e., joint hypermobility or multiple dislocations, easy bruising, poor wound healing) are frequently observed in patients with sCeAD (50%–96%) (11). Therefore, the prevailing idea is that the disease might be the end phenotype of an underlying, inherited, subclinical, systemic connective tissue disorder, leading to an arterial wall weakness. However, since no reliable biomarker has been identified to detect such subclinical abnormalities, the “*connective hypothesis*” in sCeAD pathogenesis remains not definitively proven and, in clinical practice, there are currently no tools to predict which individuals are at risk of disease occurrence. Several previous analyses of skin samples taken by biopsy in patients with sCeAD examined ultrastructural connective tissue abnormalities such as aberrations of collagen and elastic fibers that are usually found in HCTD (12, 13), while other studies attempted to identify genetic aberrations, particularly in genes involved in the extracellular matrix and collagen fibril organization, based on the familial clustering of sCeAD in some cases (7). These histologic and ultrastructural, as well as genetic findings, may provide relevant information on disease pathogenesis. Therefore, we conducted a systematic review of studies exploring the hypothesis that connective tissue abnormalities might play a role in sCeAD pathogenesis through a search for dermal connective tissue aberrations. We also summarized the results of genetic analyses, if performed in the included studies.

## 2. Methods

A systematic search was conducted using the PubMed and Scopus databases, by Nested Knowledge systematic review software,^1^ with the following search keywords: “Cervical artery dissection” or “Intracranial Dissection” or “Carotid dissection” or “Vertebral Dissection” and “Skin biopsy.” We included studies from the inception to August 2023. We also conducted a manual search and requested expert recommendations to further identify any other potential articles.

We included all original articles written in English that reported the skin biopsy assessments in patients with sCeAD. We excluded the following articles: (1) case reports, (2) not sCeAD diagnosis, (3) no skin biopsy performed, (4) animal studies, (5) non-English literature, (6) review articles, (7) letters to the editor and editorial, and (8) duplicate records. Two authors (MEG and ZK) screened the titles and abstracts using these predefined criteria. The discrepancies were assessed by all authors with the full text of the articles.

After the screening and review of the articles, we extracted the most significant data to evaluate the results of these studies. The following variables were extracted from the included articles when available: study and control population, type of dissection, mean age, time since event/stroke, primary skin biopsy outcomes, and their main results.

## 3. Results

**Figure 1:**
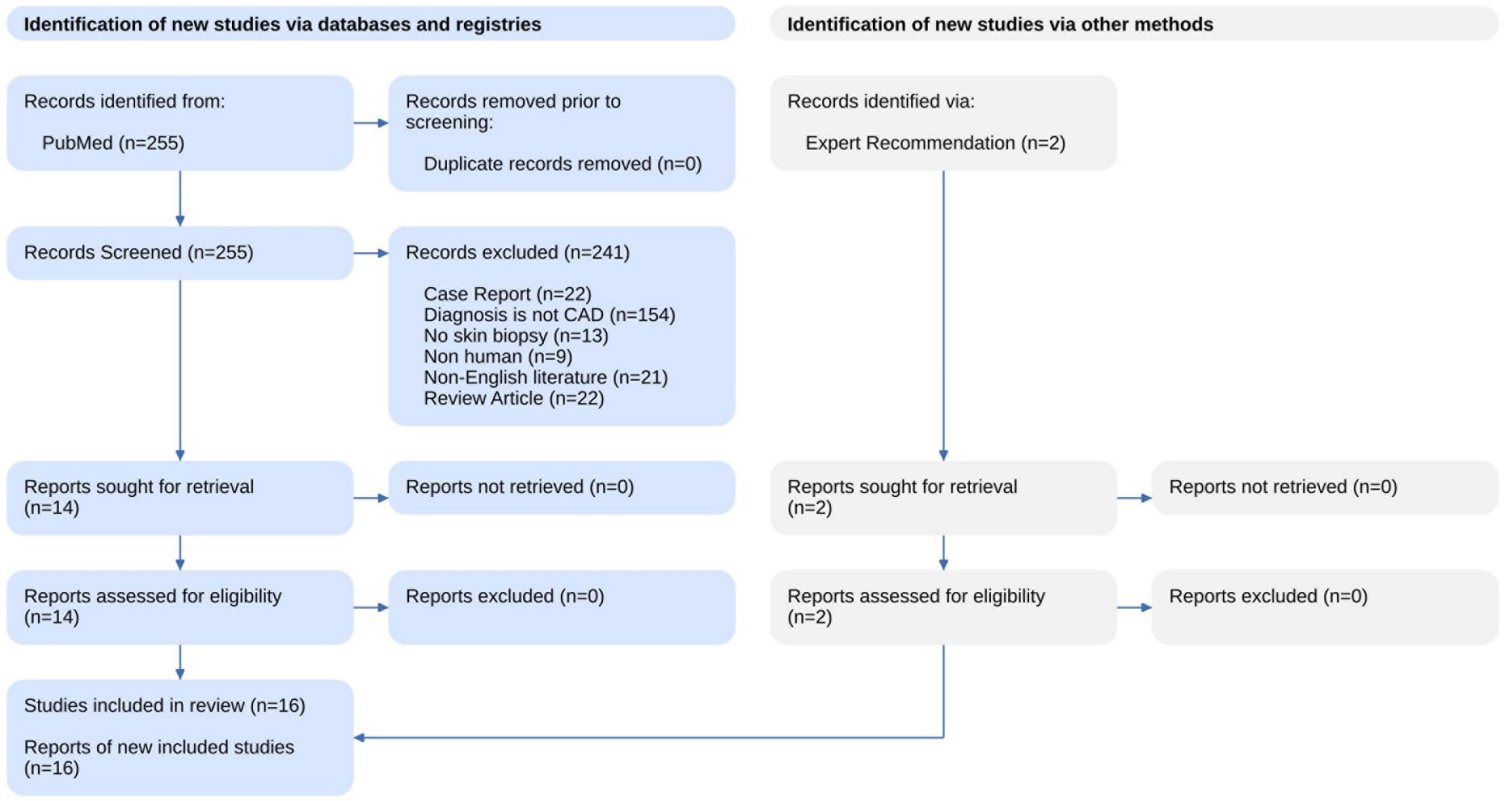
Literature search flow chart.

The results of the systematic search and summary of the screening process are available in Figure 1 as a PRISM statement flow diagram (14). Our literature search identified 255 studies from PubMed and Scopus. We excluded 241 articles based on a review of the title and abstract with the criteria listed above. Two additional studies were identified with an expert recommendation. The remaining 16 articles were assessed with full text and included in this review.

### 3.1. Demographics and clinical features

Fifteen of the studies included were case–control (15–22) and case-series studies (12, 13, 23–27) and one study was a cohort study (28). The demographic and clinical characteristics of subjects included in these 16 studies are summarized in Tables 1, 2. The aggregate number of patients included in this review was 459 (range, 3–126). All studies included sCeAD adult patients with single vessel, bilateral or multivessel involvement, and they were compared to healthy controls, other than two studies without a control group (12, 24). Fifteen studies reported ages ranged from 18–70, but most of the patients were young adults with a mean age of 41.5 ± 9.3 years. The time since the event/stroke was mostly not reported. All studies evaluated possible underlying connective tissue disorders by analysis of skin samples obtained from biopsy, in which histologic and/or ultrastructural changes were searched for, as well as by genetic analysis for possible gene involvement. Three studies investigated families with sCeAD patients, for a total of 11 families with 19 sCeAD patients (24–26).

**Table 1:** Summary of studies assessing structural aberrations in connective tissue.

References	Type of article	Sample Size	Type of dissection	Control	Age (Mean)	Primary Outcomes	Main Results
Brandt et al. (13)	Case series	25 sCeAD	ICA (14), Bilateral ICA (2), VA (8), Bilateral VA (1)	10 healthy controls	42	Blinded qualitative light and electron microscopy analysis	Collagen aberrations in 15 patients; composite fibrils with a variable diameter and a flower-like cross-section
Brandt et al. (16)	Case control	65 sCeAD	ICA (29), VA (7), Multivessel (22), Recurrent (7)	10 healthy controls	41	Ultrastructural morphology by transmission electron microscopy	Ultrastructural aberrations in 55% of patients Only 5% had clinical manifestations Recurrent sCeAD correlated with connective tissue aberrations.
Grond-Ginsbach et al. (24)	Case series	6 family members of 1 sCeAD patient	Left ICA	No control	Index patient: 48	Ultrastructural morphology by electron microscopy	Index patient and 3 children with connective tissue aberrations of collagen and elastic fibers.
Hausser et al. (19)	Case control	126 sCeAD	Not reported	29 healthy controls	Not reported	Connective tissue morphology by electron microscopic	Aberrant morphology of collagen and elastic fibers in 57% of patients
Ulbricht et al. (12)	Case series	7 sCeAD	ICA (6), VA (1)	No control	39	Ultrastructural morphology	Alterations of the collagen and elastic fibers in 6 patients
Martin et al. (25)	Case series	7 families with 15 sCeAD patients	ICA (10), VA (5)	No control	36.2	Connective tissue morphology	EDS-III and IV-like changes in 4 patients, normal in 9 patients.
Wiest et al. (26)	Case series	Families of 3 sCeAD patients	ICA (3)	No control	48	Connective tissue phenotypes by electron microscopic	All 3 families with same EDS-III like connective tissue alterations with “flower-like” composite fibrils in some collagen bundles and with fragmentation of the elastic fibers.
Völker et al. (20)	Case control	20 sCeAD	ICA (10), VA (6), ICA + VA (4)	18 (14 healthy, 4 autopsy)	41	Connective tissue morphology	Significantly smaller mean diameter of collagen fibrils in sCeAD patients No significant difference in fibril density and relative fibril area
Uhlig et al. (21)	Case control	31 sCeAD	ICA (18), Bilateral ICA (1), VA (7), Bilateral VA (2), Multivessel (3)	17 healthy controls	40.8	Collagen fibril abnormalities by transmission electron microscopy	20% of sCeAD patients showed collagen fibril alterations; irregularly contoured surfaces and increased diameters, often associated with a faint or absent banding pattern
Erhart et al. (27)	Case series	4 sCeAD patients with additional dissection in other vasculature beds	1. Right ICA, aorta type B 2. Bilateral ICA, left VA, aorta type B 3. Left ICA, aorta type A 4. Right ICA, aorta type B	No control	41.7 ± 5.1	Ultrastructural morphology by electron microscopy	3 patients had morphologic alterations of the dermal connective tissue (small-caliber and composite/abnormal collagen fibrils)

ICA, internal carotid artery; VA, vertebral artery; EDS: Ehlers-Danlos syndrome.

**Table 2:** Summary of studies with genetic analyses from skin biopsy.

References	Type of article	Sample size	Type of dissection	Control	Age (mean)	Primary outcomes	Main results
van den Berg et al. (15)	Case control	16 sCeAD	ICA (10), Bilateral ICA (3), VA (3)	41 healthy controls	40.7	Protein analysis of type III collagen	No mutation in the gene of type III collagen was demonstrated.
Grond-Ginsbach et al. (23)	Case series	10 sCeAD	ICA (5), VA (2), Recurrent (3)	1 healthy control	44	Gene encoding tropoelastin (ELN) sequence analysis	No mutations in the whole coding region of the ELN gene
von Pein et al. (17)	Case control	12 sCeAD	Partially reported	50 healthy controls	42.5	Sequence analysis of the COL3A1 gene	No disease-causing mutations in the COL3A1 in gene.
Morcher et al. (18)	Case control	12 sCeAD	Partially reported	25 healthy controls	38.8	Genomic sequencing of the ABCC6 gene	No changes in ABCC6 gene.
Martin et al. (25)	Case series	7 families with 15 sCeAD patients	ICA (10), VA (5)	203 healthy subjects	36.2	Coding sequences of COL3A1, COL5A1, COL5A2, and COL1A1	Only a missense mutation in the COL3A1 gene.
Wiest et al. (26)	Case series	Families of 3 sCeAD patients	ICA (3)	No control	48	Genome-wide linkage analysis	Linkage between sCeAD-associated connective tissue alterations and chromosome 15q2 and 10q26 mutations Locus heterogeneity in connective tissue phenotype of sCeAD patients
Uhlig et al. (21)	Case control	31 sCeAD	ICA (18), Bilateral ICA (1), VA (7), Bilateral VA (2), Multivessel (3)	17 healthy controls	40.8	Collagen fibril abnormalities by transmission electron microscopy	20% of sCeAD patients showed collagen fibril alterations; irregularly contoured surfaces and increased diameters, often associated with a faint or absent banding pattern
Grond-Ginsbach et al. (22)	Case control	70 sCeAD	Not reported	403 controls	42.5 ± 9.8	CNVs screening and Gene Ontology analysis	Significant CNVs enrichments for genes involved in Extracellular matrix (COL5A2, COL3A1, SNTA1) and collagen fibril (COL5A2, COL3A1) organizations
Mayer-Suess et al. (28)	Cohort Study	38 sCeAD	Single vessel (19), Multivessel (13), Recurrent (6)	12 healthy controls	49.1	Extracellular Matrix Protein analysis from skin punch biopsies using quantitative proteomics approach	No difference in single-vessel or multiple-vessel dissections between each other or compared to healthy controls Recurrent sCeAD showed significantly different expression of 25 proteins compared to the other groups combined. 13 proteins were linked to connective tissue disorders.
Erhart et al. (27)	Case series	4 sCeAD patients with additional dissection in other vasculature beds	1. Right ICA, aorta type B 2. Bilateral ICA, left VA, aorta type B 3. Left ICA, aorta type A 4. Right ICA, aorta type B	No control	41.7 ± 5.1	Whole-exome sequencing and CNV analysis	3 patients carried pathogenic variants in COL3A1, COL5A2, and/or MYH11 genes

ICA: Internal carotid artery, VA: Vertebral artery, CNV: copy number variant.

### 3.2. Structural aberrations in connective tissue

Table 1 summarizes the findings from ten studies (12, 13, 16, 19–21, 24–28) that evaluated the connective tissue morphology showed ultrastructural aberrations, in the absence of clinical manifestations, such as alteration of the collagen and elastic fiber networks (12), significantly thinner dermis (19), smaller collagen fibrils diameter in the skin (20, 27), abnormal collagen fibril structures with faint and absent banding pattern (21, 24, 27), aberrations in numerous composite fibrils within mid-dermal collagen bundles with enlarged diameters of composite fibrils (13, 24) (very similar to the aberrations seen in patients affected by EDS-II or III (12, 16)) and elastic fiber abnormalities with mini calcifications and fragmentation (16). All of these studies were consistent in showing aberrations of the connective tissue, in a cumulative number of 149 out of 295 (50.5%) patients.

### 3.3. Genetic analyses

Besides the morphological evaluation, molecular analyses that were performed in 10 out of the 15 studies did not detect any causative mutations in genes encoding for collagen type I, III, V, and elastin and sCeAD (15, 17, 18, 22–26, 28). Table 2 summarized the findings of genetic analyses from skin biopsy studies.

Notwithstanding, the linkage analysis of a large family with sCeAD-associated connective tissue alterations suggested the presence of a candidate locus on chromosome 15q2 or on chromosome 10q26 (26), while other studies revealed copy number variants (CNV) enrichment in genes involved in extracellular matrix organization (COL5A2, COL3A1, SNTA1), collagen fibril organization (COL5A2, COL3A1), smooth cell functions (MHY11) and possibly in genes involved in transforming growth factor beta (TGF)-beta receptor signaling pathway (COL3A1, DUPS22) (22, 27). Finally, Mayer-Suess and co-workers, based on the analysis of the extracellular matrix proteins by the proteomics approach, showed different expressions of 25 proteins, 13 of which were linked to connective tissue disorders, in patients with recurrent sCeAD (28).

Overall, although the results of the genetic analyses included in this systematic review do not allow for definitive conclusions, they seem to support the hypothesis of a potential involvement of “connective genes” in sCeAD pathogenesis.

## 4. Discussion

In this review, we summarized the results of the studies examining connective tissue abnormalities through the analysis of samples obtained by skin biopsy in patients with sCeAD. Given the lack of biologic or molecular markers specifically linked to the disease, histologic and ultrastructural connective tissue abnormalities detected by the analysis of skin samples potentially represent a useful tool to indirectly support the hypothesis that structural and functional alterations of the arterial connective component may play a crucial role in the pathogenesis of sCeAD. This is made even more biologically plausible when we consider that these connective tissue elements provide mechanical stability and are responsible for most of the functional properties of the arterial wall.

### 4.1. Evidence from the analysis of skin biopsies

Our review showed that alterations of the collagen and elastic fiber networks are frequent findings in patients with sCeAD as opposed to what is observed in subjects without sCeAD. Such dermal connective tissue aberrations include irregular (flower-like) contours with variable diameters of collagen fibrils, and fragmented, moth-eaten-like appearance and microcalcifications of elastic fibers. These alterations are similar to those that may be found in classic HCTD such as in Ehlers–Danlos syndrome type IV or pseudoxanthoma elasticum, a further argument in favor of the “connective hypothesis” of sCeAD.

### 4.2. Evidence from the genetic analyses

Over the last decades, it has been repeatedly emphasized that classical HCTD, such as EDS type IV and Marfan syndrome, or other more recently identified nosographic entities, such as the Loeys-Dietz syndrome (LDS) comprise sCeAD in their phenotypic spectrum. However, diagnostic criteria for HCTD are met in only 1%–5% of sCeADs patients (10, 29). This implicates that there might be other unrecognized connective tissue abnormalities predisposing the vessel wall to dissection. The results of the studies included in the present review support this hypothesis. First, a family history of CeAD in some cases indirectly suggests that genetic factors might be operant in the pathogenesis of the disease. This hypothesis was further supported by the observation that dermal ultrastructural connective abnormalities not fulfilling the diagnostic criteria for known HCTD (26) aggregate in familial groups in some cases, where they follow an autosomal-dominant pattern of inheritance.

Second, besides searching for morphological aberrations, molecular analyses were also conducted in some of the studies included in our systematic review. The majority of these studies focused on the genes of collagen type I (COL1A1) (25), type III (COL3A1) (15, 17, 25), type V (COL5A1, COL5A2) (23, 25) as well as elastin (ELN) (23), but they were unable to identify any causative mutations in sCeAD. Although collagen type III was particularly an area of interest as the mutation in this gene is related to EDS type IV (the “vascular” subtype of EDS), none of these studies identified causative mutations in this gene among patients with sCeAD other than the missense mutation G157S in two patients from the same family. The major limitation of these studies is inherent in their nature of small case series, which makes them not fully adequate for determining the prevalence of such causative mutations among sCeAD patients.

Similarly, most of the studies in which a genetic linkage analysis was performed gave negative results, with the only exception of one study suggesting a linkage between connective tissue alterations and mutations in genes involved in extracellular matrix and collagen fibril organization, especially in patients with recurrent sCeAD, in whom a greater pathogenic impact of structural vessel wall alterations is assumed.

More advanced methods such as whole-exome and whole-genome sequencing have been recently used to investigate patients with sCeAD. One study performed a genome-wide linkage analysis and found suggestive linkage between CAD-associated connective tissue alterations and mutation of the locus on chromosome 15q2 and chromosome 10q26 (26). This approach identified several promising candidate genes such as CSPG2, LOXL1, and FGFR2, all already known to be involved in aortic dissections and aneurysms formation.

Another study searched for rare genetic deletions and duplications that predispose to sCeAD based on the analysis of CNV (22). This study showed significant CNV enrichments in genes involved in extracellular matrix organization (COL5A2, COL3A1, SNTA1) and collagen fibril organization (COL5A2, COL3A1). Interestingly, none of these rare CNV enrichments were found in more than one patient, which indicated that the underlying genetic variation is a complex and heterogeneous process that cannot be explained with simple monogenetic variations. Furthermore, one small study performed whole-exome sequencing and CNV analysis in patients with independently occurring dissections in both the aorta and cervical arteries, and similarly identified pathogenic CNV in COL3A1, COL5A2, and MYH11 genes, which all involved in arterial connective tissue functions (27).

Finally, a recent study used a cutting-edge quantitative proteomics approach to identify extracellular protein aberrations in sCeAD patients and identified 25 proteins expressed differently only in patients with recurrent sCeAD (28). The Authors also identified two main protein clusters; (1) desmosome-associated cluster with four proteins and (2) collagen and elastin cluster with eight proteins, suggesting a more complex pathophysiology of sCeAD and possibly more evident aberrations at the proteome level. These results suggest underlying pathological genetic variants, particularly in patients with recurrent and multiple territory dissections.

### 4.3. Further indirect evidence of relationship between sCeAD and connective tissue disorder

The literature suggests further arguments in favor of the “connective hypothesis” in the pathogenesis of sCeAD.

First, echocardiographic studies assessing cardiac manifestations of connective tissue disorder showed that valvular abnormalities such as mitral valve prolapse, mitral valve dystrophy, aortic valve dystrophy as well as the enlarged diameter of the aortic root were observed more frequently in sCeAD patients (56%) compared to controls (15%) (30). One study, in particular, showed that aortic root dilatation, one of the classical signs of HCTD, particularly seen in Marfan syndrome or Ehlers-Danlos syndrome, was strongly associated with sCeAD.

Second, studies investigating arterial wall biomechanical proprieties in sCeAD patients found altered arterial distensibility. Guillon and co-workers, in an ultrasound study focusing on common carotid artery diameter and diameter changes during the cardiac cycle showed that the diameter changes were more pronounced in sCeAD, reflecting less elastic properties of the arterial wall (31). Similarly, in another study Calvet and co-workers assessed the elastic properties of the carotid wall in sCeAD patients using noninvasive high-resolution echo tracking systems and showed that sCeAD patients had higher circumferential wall stress and stiffness (32). Besides, this study also showed that Young’s modulus, a direct measure of elasticity proprieties of the vessel, was significantly higher (indicating stiffer arterial wall), and associated with an up to the 8-fold increased risk of sCeAD. Lucas and co-workers assessed endothelial function and reactivity using high-resolution ultrasonography and showed that the brachial artery flow-mediated vasodilatation was significantly reduced in patients with sCeAD compared to controls, indicating impaired endothelial-dependent vasodilation (33).

Third, weakened arterial structure caused by connective aberrations may also predispose to vascular deformities, such as tortuosity. Higher arterial tortuosity was associated with connective tissue diseases, particularly Marfan syndrome and Loeys-Dietz syndrome (34, 35). In a study using magnetic resonance angiography Giossi and co-workers showed that tortuosity indexes in sCeAD patients were significantly higher compared to those of matched controls (36). These findings are indirectly supported by the observation that aortic dissection in patients affected by Marfan syndrome correlates with arterial tortuosity of the aorta (37).

Fourth, Brandt and co-workers showed that the ultrastructural connective tissue abnormalities in collagen and elastin fibrils of patients with sCeAD were related to disease recurrence (16). They also showed that these aberrations were more commonly seen in male patients but were not associated with age and vascular risk factors.

Fifth, indirect evidence in favor of the connective hypothesis also comes from studies investigating clinically detectable connective signs in sCeAD patients. Dittrich and co-workers assessed the clinical connective tissue phenotype of sCeAD patients using a standardized examination containing 25 clinical items that mainly included signs found in Marfan and Ehlers-Danlos syndromes (38). Presumably due to the fact that the sample size was relatively small to detect small clinical changes, they found no significant difference in clinically detectable connective tissue abnormalities in patients with sCeAD compared with the non-sCeAD group. Conversely, another more recent study with a larger sample size assessed clinically detectable connective tissue abnormalities with a more extensive standardized examination protocol including 68 items found in HCTD (11) and showed a higher prevalence of connective tissue abnormalities in sCeAD patients, further supporting the connective hypothesis. Interestingly, the low prevalence of these clinically detectable signs in patients with traumatic CeAD contrary to sCeAD further supports this hypothesis. Similarly to previous studies discussed in this review, these patients were not diagnosed with a definite HCTD, implicating that sCeAD may represent a multifactorial disease that is the result of the combination of triggering factors in settings of an underlying subclinical connective tissue disorder.

## 5. Conclusion and future directions

Data reported in this review support the presence of an underlying subclinical, likely genetically determined, connective tissue disorder predisposing to arterial wall weakness and sCeAD. Our current knowledge is limited by the small sample size, the specific design of some studies, the cost-resource requirements of these methods, as well as by the characteristics of the disease itself, the pathogenesis of which is likely to be complex. Further studies with larger sample sizes and robust methodology are needed to better define the role of connective tissue in sCeAD pathogenesis. Although the current evidence is not enough to change the current guidelines, a better understanding of the connective tissue aberrations and the underlying genetic features is relevant to identifying people at risk of developing sCeAD. Skin biopsy and genetic testing should be considered particularly for patients with recurrent sCeAD and dissections in multiple vasculature beds. This is also important to develop future therapies targeting vessel wall strength aiming to prevent sCeAD occurrence and/or its complications such as disease recurrence and dissecting pseudoaneurysm formation. This may allow individualizing medical and endovascular treatments.

## Data availability statement

The original contributions presented in the study are included in the article/supplementary material, further inquiries can be directed to the corresponding author.

## Author contributions

MG and ZK independently screened the titles and abstracts for systematic review inclusion. MG, RK, DK, AP, and ZK involved in assessing the discrepancies with the full text of the article and the manuscript writing. ZK had final responsibility for the decision to submit for publication. All authors contributed to the article and approved the submitted version.

## Footnotes

^1^https://nested-knowledge.com/
